# Long-Term Osteoporosis Risk in Colorectal Cancer Survivors: A Nationwide Longitudinal Cohort with up to 16 Years of Follow-Up

**DOI:** 10.3390/biomedicines13092159

**Published:** 2025-09-04

**Authors:** Ho Suk Kang, Joo-Hee Kim, Eun Soo Kim, Dae Myoung Yoo, Kyeong Min Han, Nan Young Kim, Hyo Geun Choi, Ha Young Park, Mi Jung Kwon

**Affiliations:** 1 Division of Gastroenterology, Department of Internal Medicine, Hallym University Sacred Heart Hospital, Hallym University College of Medicine, Anyang 14068, Republic of Korea; hskang76@hallym.or.kr; 2 Division of Pulmonary, Allergy, and Critical Care Medicine, Department of Medicine, Hallym University Sacred Heart Hospital, Hallym University College of Medicine, Anyang 14068, Republic of Korea; luxjhee@gmail.com; 3 Department of Radiology, Hallym University Sacred Heart Hospital, Hallym University College of Medicine, Anyang 14068, Republic of Korea; silwater007@hallym.or.kr; 4 Hallym Data Science Laboratory, Hallym University College of Medicine, Anyang 14068, Republic of Korea; ydm1285@naver.com (D.M.Y.); km_han@hallym.ac.kr (K.M.H.); 5 Hallym Institute of Translational Genomics and Bioinformatics, Hallym University Medical Center, Anyang 14068, Republic of Korea; honeyny78@gmail.com; 6 Suseo Seoul E.N.T. Clinic, 10, Bamgogae-ro 1-gil, Gangnam-gu, Seoul 06349, Republic of Korea; mdanalytics@naver.com; 7 Department of Pathology, Busan Paik Hospital, Inje University College of Medicine, Busan 47392, Republic of Korea; hy08.park@gmail.com; 8 Department of Pathology, Hallym University Sacred Heart Hospital, Hallym University College of Medicine, Anyang 14068, Republic of Korea

**Keywords:** colorectal cancer, osteoporosis, risk assessment, nationwide health insurance research database, longitudinal follow-up study

## Abstract

**Background/Objectives:** Colorectal cancer (CRC) survivors may face long-term health consequences, yet the relationship between CRC and osteoporosis remains underexplored, particularly in Asia. We conducted a nationwide, retrospective longitudinal cohort study with matched controls using the Korean National Health Insurance Service–National Sample Cohort (2005–2019) to assess whether CRC increases osteoporosis risk or not. **Methods:** We identified 8733 CRC patients and 34,932 matched controls (1:4 ratio) based on age, sex, income, residence, and index date, excluding individuals with pre-existing osteoporosis. Osteoporosis was defined via ICD-10 codes (M80–M82) plus confirmatory bone imaging claims. Propensity score overlap weighting was applied, and adjusted hazard ratios (HRs) with 95% confidence intervals (CIs) were calculated using Cox proportional hazards models, with subgroup analyses based on demographic and clinical factors. **Results:** With up to 16 years of follow-up, osteoporosis incidence rates were 13.80 and 14.30 per 1000 person-years in CRC and control groups, respectively. Adjusted Cox models revealed no significant association between CRC and osteoporosis (HR = 0.95; 95% CI = 0.87–1.04). Subgroup analysis showed a slightly lower risk among CRC survivors aged ≥65 years (adjusted HR = 0.84; 95% CI = 0.75–0.95), though no associations were observed by sex, income, region, or comorbidities. **Conclusions:** These findings suggest CRC may not be an independent risk factor for osteoporosis in the Korean population. The unexpected age-specific variation warrants cautious interpretation, possibly reflecting competing mortality risks or detection biases.

## 1. Introduction

Colorectal cancer (CRC) is among the most common malignancies globally, ranking third in incidence and second in cancer-related mortality, and accounts for nearly one-tenth of all cancer diagnoses and fatalities [1]. In 2018, South Korea reported the world’s second-highest CRC incidence rate—44.5 new cases per 100,000 individuals annually—representing a substantial national health burden [2]. Advances in CRC screening, treatment, and public health programs have improved five-year survival rates, creating a growing population of long-term survivors [3]. As recurrence surveillance remains a priority, there is a growing need to address long-term complications and comorbidities [4,5].

Osteoporosis—a progressive bone disorder involving decreased mineral density and compromised microarchitecture, increasing fracture risk—is an important concern in aging populations and is emerging as a significant long-term health burden in cancer survivors [6,7]. Osteoporosis is well recognized as a complication of hormonal therapy in breast and prostate cancer, where long-term endocrine treatments substantially increase bone loss and fracture risk [8,9]. However, CRC survivors represent a distinct and under-investigated group. CRC is one of the most common malignancies worldwide, with rapidly improving survival rates owing to advances in screening and therapy, resulting in a growing population of long-term survivors [1,3,4]. Unlike breast and prostate cancer, where osteoporosis risk may be primarily driven by hormone deprivation, CRC survivors are likely to be exposed to different treatment modalities—including surgery, corticosteroid use, and pelvic radiotherapy—that may uniquely affect bone health [4,10,11]. Moreover, CRC and osteoporosis share several non-treatment-related risk factors, such as aging, systemic inflammation, poor nutrition, and inactivity [4]. Disruption of calcium and vitamin D metabolism may be observed in CRC patients [4,6,9]. In the Korean population, osteoporosis has been identified as an independent predictor of colorectal adenoma, suggesting shared biological pathways between low bone mass and developing colorectal neoplasia, such as chronic inflammation, altered calcium and vitamin D metabolism, and hormonal regulation [12]. However, the reverse association—CRC as a risk factor for osteoporosis—has not been reported in the Korean population.

Despite this biological plausibility [4], there is a paucity of large-scale, population-based studies evaluating the relationship between CRC and osteoporosis. To date, only one cohort study—the U.S. Medicare-linked SWOG trial by Barzi et al. [6]—has specifically examined osteoporosis incidence among CRC survivors, reporting a 4.85-fold higher risk in female survivors compared with male survivors (95% CI = 2.14–3.93) and a 2.90-fold higher risk compared with the general U.S. population (95% CI = 2.14–3.93). These findings highlight the need for further research, particularly in Asian populations, to validate osteoporosis as a significant comorbidity in CRC survivors and to characterize its longitudinal risk using nationally representative data.

In this study, we hypothesized that CRC is positively associated with the risk of developing osteoporosis and that this association may vary according to individual characteristics such as age, sex, socioeconomic status, and comorbid conditions. Our primary objective was to investigate the relationship between CRC and osteoporosis risk, which is an important and underrecognized public health issue in long-term outcomes in CRC survivors. We also explored whether lifestyle or demographic factors—including age, sex, smoking status, and rural residence—modify this risk. Using longitudinal data from the Korean national public healthcare system, we analyzed this association while adjusting for potential confounders.

## 2. Materials and Methods

### 2.1. Research Design, Data Resource, and Cohort Selection

This retrospective longitudinal cohort study with matched controls was approved by the Institutional Review Board of Hallym University Sacred Heart Hospital (IRB No. 2022-10-008), with informed consent waived due to the use of de-identified data. All study procedures adhered to relevant ethical standards.

Data were obtained from the Korean National Health Insurance Service–National Sample Cohort (KNHIS-NSC), which was constructed in 2002 through systematic sampling to represent approximately 2.2% of the Korean population [13]. Participants were followed for up to 18 years, until 2019, unless follow-up ended due to death or emigration. Detailed descriptions of the database structure and representativeness have been provided in previous publications [14].

CRC cases were identified from 2005 to 2019 based on the simultaneous presence of both an International Classification of Diseases, 10th Revision (ICD-10) diagnostic code—C18 (colon), C19 (rectosigmoid junction), or C20 (rectum)—and a national cancer-specific reimbursement code (V193 or V194). These reimbursement codes are assigned only to pathologically confirmed cancer patients registered in the national cancer registry to receive financial support for critical illnesses. This dual-coding approach, widely used in Korean administrative database research, enhances diagnostic specificity and reduces misclassification by excluding suspected or provisional cases [15]. Individuals with only a diagnostic code or only a reimbursement code were excluded.

The initial control pool included individuals without any CRC diagnosis or reimbursement code between 2005 and 2019 (*n* = 1,127,941). To improve diagnostic accuracy, we excluded 3472 individuals who had any CRC-related code recorded at least once. Participants with a history of osteoporosis prior to the index date were also excluded, resulting in the removal of 1187 individuals from the CRC group.

To minimize baseline differences, we first performed 1:4 exact matching between CRC patients and controls by age, sex, income level, and residential area. The index date—defined as the first date on which both a CRC diagnostic code and reimbursement code were recorded—was assigned to the matched control in each pair. Following matching, we estimated propensity scores using all baseline covariates and applied overlap weighting to further balance residual differences and optimize comparability. This approach emphasizes the region of common support between groups, retains all participants, and approximates the balance achieved in randomized controlled trials. During matching, 1,089,537 controls were excluded due to unmatched characteristics, yielding a final study population of 8733 CRC patients and 34,932 matched controls.

**Figure 1:**
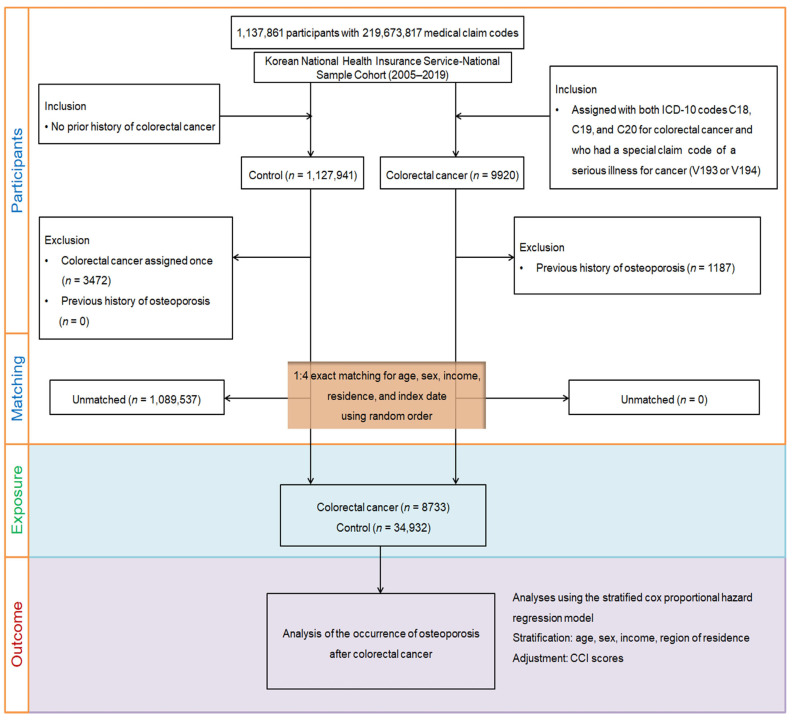
Flowchart of participant selection. From the Korean National Health Insurance Service–National Sample Cohort, colorectal cancer (CRC) cases were identified by concurrent ICD-10 codes C18–C20 and cancer-specific reimbursement codes (V193 or V194) between 2005 and 2019. Individuals with prior osteoporosis, incomplete baseline data, or unmatched characteristics were excluded. After 1:4 exact matching on age, sex, income, and residence, and applying propensity score overlap weighting, the final cohort included 8733 CRC patients and 34,932 matched controls. Participants were followed for incident osteoporosis until diagnosis, death, or 31 December 2019.

Osteoporosis was defined using ICD-10 codes M80 (osteoporosis with pathological fracture), M81 (osteoporosis without pathological fracture), and M82 (osteoporosis associated with other diseases) [16,17]. To increase diagnostic validity, only individuals with at least two clinical visits supported by bone mineral density testing via X-ray or computed tomography (procedure codes HC341–HC345, E7001–E7004) were considered incident cases [16,17]. Follow-up continued from the index date until the earliest occurrence of osteoporosis diagnosis, death, or 31 December 2019 (Figure 1).

### 2.2. Covariates and Comorbidity Adjustment

Covariates included age (classified into 18 groups at 5-year intervals), income level (five categories from lowest to highest), and residential area (urban or rural, per our previous classification method). Comorbidity burden was assessed using the Charlson Comorbidity Index (CCI), which assigns scores from 0 to 29 for the presence and severity of 17 chronic conditions (excluding cancer), including myocardial infarction, heart failure, peripheral vascular disease, cerebrovascular disease, dementia, chronic pulmonary disease, connective tissue disorders, peptic ulcer disease, liver disease, diabetes (with or without complications), paraplegia, renal disease, metastatic cancer, severe liver disease, and HIV/AIDS [18]. Given the potential for comorbidities to confound associations between CRC and osteoporosis likelihood, we adjusted for CCI scores and individual comorbidities as covariates in our analyses. To further reduce residual confounding and enhance comparability, we employed overlap weighting alongside multivariable Cox-proportional hazards models.

### 2.3. Statistical Analyses

Categorical variables were summarized as frequencies and percentages, and continuous variables as means with standard deviations. Group balance was evaluated using standardized differences, with values below 0.20 indicating adequate balance. Incidence rates (IRs) and incidence rate differences (IRDs) were calculated per 1000 person-years. Cumulative incidence was assessed using the Kaplan–Meier method with the log-rank test, and hazard ratios (HRs) with 95% confidence intervals (CIs) were estimated using Cox proportional hazards models. The proportional hazards assumption was verified before model interpretation. Subgroup analyses were performed according to demographic and clinical characteristics, including age, sex, income level, residential area, and CCI score. All statistical analyses were conducted using SAS version 9.4 (SAS Institute Inc., Cary, NC, USA), and two-sided *p*-values < 0.05 were considered statistically significant.

## 3. Results

A total of 43,665 participants were included in the analysis, comprising 8733 patients with CRC and 34,932 matched controls. Matching was performed in a 1:4 ratio based on age, sex, income level, and residential area, resulting in standardized differences of 0.00 for these variables, indicating complete balance between groups. Other baseline characteristics, including CCI scores and the post-index incidence of selected comorbidities, showed minimal differences (standardized differences = 0.10 and 0.07, respectively) (Table 1).

**Figure 2:**
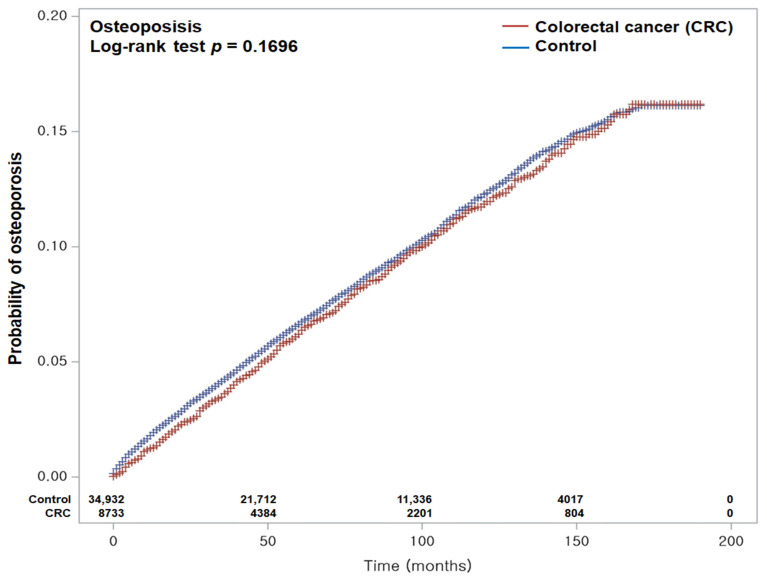
Kaplan–Meier curves showing the cumulative incidence of osteoporosis in patients with colorectal cancer (CRC) and matched controls with up to 16 years of follow-up from the index date. The log-rank test revealed no statistically significant difference in osteoporosis incidence between the CRC and control groups throughout the study period.

During the follow-up period, osteoporosis occurred in 579 CRC patients (6.63%) and 2909 controls (8.33%). The corresponding incidence rates were 13.80 and 14.30 per 1000 person-years, respectively. Both crude and adjusted HRs indicated no significant difference in osteoporosis risk between the CRC and control groups (crude HR = 0.96, 95% CI: 0.87–1.05, *p* = 0.330; adjusted HR = 0.95, 95% CI: 0.87–1.04, *p* = 0.302) (Table 2). Kaplan–Meier curves and log-rank testing similarly revealed no significant difference in cumulative osteoporosis incidence between groups (*p* = 0.170; Figure 2).

Subgroup analyses stratified by age, sex, income level, residential area, and CCI score demonstrated generally consistent findings, with no significant association between CRC and osteoporosis risk in most strata (Table 2). Notably, CRC patients aged ≥ 65 years exhibited a modest but statistically significant reduction in osteoporosis risk compared with matched controls (adjusted HR = 0.84, 95% CI: 0.75–0.95, *p* = 0.005). No significant associations were observed in other subgroups, including patients aged < 65 years.

## 4. Discussion

In this large, nationwide long-term follow-up study, CRC was not associated with an increased risk of osteoporosis in the Korean population, even after adjusting for potential confounders such as age, comorbidities, and socioeconomic status, indicating that CRC may not serve as an independent risk factor for osteoporosis. Thus, our overall results did not support the initial hypothesis that CRC survivors would have a higher risk of osteoporosis; in other words, the null hypothesis of no association could not be rejected in the general population. These results are partially supported by large prospective, double-blind randomized trials of anti-osteoporotic drugs, including bisphosphonates, which have not demonstrated any relationship with gastrointestinal cancers—including CRC—even with follow-up periods of up to 10 years [19,20,21]. Likewise, a meta-analysis reported no significant difference in osteoporosis incidence between cancer survivors and healthy counterparts [22].

In contrast, several Western studies have reported an increased osteoporosis risk among CRC survivors. The U.S. Medicare-linked SWOG trial by Barzi et al. [6] found a 4.85-fold higher risk of osteoporosis among female CRC survivors compared with their male counterparts (95% CI = 2.14–3.93) and a 2.90-fold higher risk compared with the general U.S. population (95% CI = 2.14–3.93), indicating a pronounced sex disparity. This was the first large, population-based study to highlight osteoporosis as a significant comorbidity in CRC survivors. Similarly, a UK study using the General Practice Research Database reported a 41% higher osteoporosis risk in CRC patients than in non-cancer controls (95% CI = 1.15–1.73) [9]. However, both studies included other cancers (e.g., breast, prostate) and did not focus exclusively on CRC, limiting cancer-specific interpretation. Furthermore, osteoporosis in cancer patients is most commonly linked to hormone therapy-related cancers, such as breast and prostate cancer [8,9,23]. Differences in country settings, outcome definitions, covariate adjustments, underlying population characteristics, and sample sizes likely account for these discrepancies [24,25]. Importantly, both Western studies lacked robust matching or adjustment for demographic, socioeconomic, and clinical confounders, potentially introducing bias. Their relatively small sample sizes and marked sex imbalance in osteoporosis screening among CRC patients (men *n* = 739 vs. female *n* = 494 [6]; men *n* = 2569 vs. female *n* = 2499 [9]) further suggest baseline disparities [26]. In the SWOG trial, female CRC survivors constituted 83% of cases [6]. Such demographic imbalances are likely responsible for substantial differences in study population composition [24,26], and observed heterogeneity in sex-specific outcomes may reflect intrinsic differences in the underlying characteristics of research cohorts [27]. These limitations may restrict the ability to fully evaluate osteoporosis as a comorbidity in CRC survivors.

In contrast, our Korean cohort showed no clear sex-based trend, possibly reflecting not only genetic susceptibility but also broader contextual factors [1,3,4]. These may include differences in treatment protocols (e.g., pelvic radiotherapy utilization, chemotherapy regimens), dietary patterns and nutritional intake, lifestyle behaviors such as smoking and physical activity, as well as healthcare utilization and screening practices between Western and East Asian populations [4,10,11]. Such factors, in addition to sample size and racial variation, may contribute to the discrepant findings across populations [6,9,19,20,21,22]. To limit selection bias and improve precision, we created a balanced cohort of 8733 CRC patients and 34,932 non-CRC controls matched 1:4 by age, sex, income, residence, and index date, with overlap-weighting to further balance baseline covariates, which could imitate randomized experiments [28]. This approach allowed for a well-founded, long-term comparison, ultimately showing no increased overall osteoporosis risk in CRC survivors.

Interestingly, a slightly reduced osteoporosis risk was observed among CRC survivors aged over 65 years. This finding should be interpreted cautiously, as it may be influenced by competing risks (e.g., higher mortality), reduced screening in the control population, or increased medical surveillance in cancer survivors. More frequent follow-up could lead to earlier detection and intervention, including lifestyle changes and pharmacologic treatments [29] that may help reduce osteoporosis risk. A cancer diagnosis may also prompt healthier behaviors—such as increased physical activity and cessation of smoking or alcohol—that benefit bone health [30]. Other possible explanations include improved post-treatment nutritional support, a lower proportion of patients receiving bone-affecting therapies, or higher baseline bone mineral density before cancer diagnosis, which could confer resilience to post-treatment bone loss [31,32].

Genetic susceptibility to osteoporosis, along with population-specific bone health characteristics, should also be considered when interpreting these findings. For example, Koreans generally have lower bone mineral density than Western populations [33], which may partly explain higher screening and diagnostic rates for osteoporosis in Korea, potentially introducing indication bias. Compared with U.S. participants in the 2007–2010 NHANES, bone mineral density at all skeletal sites in both Korean men and women was significantly lower [33,34]. Additionally, Korean women aged 20–49 years had lower lumbar spine and femur bone mineral density than Japanese women [33,34]. Europeans also tend to have higher bone mineral density than Chinese populations, though these differences disappear after adjusting for height [33,34].

This study has several strengths, including its use of a large, nationally representative cohort, long-term follow-up, and comprehensive statistical techniques that closely approximate randomized trial conditions [28]. The large sample size was not determined by a priori power calculation but was derived from the full availability of the KNHIS-NSC, which includes all eligible CRC patients and matched controls. While a large cohort naturally increases the likelihood of detecting statistically significant results, our interpretation emphasized effect sizes and confidence intervals rather than statistical significance alone, to ensure that findings reflect clinically meaningful associations.

Nevertheless, several limitations should be acknowledged. First, the study relied on claims-based data, which may introduce misclassification or underdiagnosis of both colorectal cancer and osteoporosis despite the use of dual-coding strategies and confirmatory imaging claims. Second, residual confounding cannot be completely excluded, as important lifestyle factors such as diet, smoking, alcohol intake, physical activity, and body mass index were not available in the dataset. Third, detailed clinical information, including cancer stage, treatment regimens, and bone mineral density measurements, was not captured, limiting assessment of stage-specific or treatment-related effects. Fourth, although propensity score weighting minimized imbalances, unmeasured variables may still bias the results. Finally, because this study was conducted within a single, relatively homogeneous East Asian population and healthcare system, generalizability to other ethnic groups or healthcare settings may be limited. Despite these limitations, the large sample size, long-term follow-up, and robust methodological adjustments strengthen the validity and relevance of our findings.

## 5. Conclusions

In summary, this large, nationwide cohort study with up to 16 years of follow-up found no significant association between colorectal cancer and subsequent risk of osteoporosis. Thus, our overall results did not support the initial hypothesis that CRC survivors would have a higher risk of osteoporosis; in other words, the null hypothesis of no association could not be rejected in the general population. The slightly lower risk observed among CRC survivors aged over 65 should be interpreted with caution, as it may be influenced by competing risks, detection bias, or differences in baseline bone health and healthcare utilization. Our findings underscore the need to account for population-specific bone health profiles and possible genetic predisposition when assessing osteoporosis risk in cancer survivors. Future studies incorporating detailed clinical, lifestyle, and genetic information are warranted to better elucidate the underlying mechanisms and guide targeted prevention strategies.

## Figures and Tables

**Table 1:** General characteristics of participants.

Characteristics		Total Participants		
		Colorectal Cancer	Control	Standardized Difference
Age (y), *n* (%)				0.00
	0–4	1 (0.01)	4 (0.01)	
	5–9	N/A	N/A	
	10–14	3 (0.03)	12 (0.03)	
	15–19	1 (0.01)	4 (0.01)	
	20–24	8 (0.09)	32 (0.09)	
	25–29	26 (0.30)	104 (0.30)	
	30–34	93 (1.06)	372 (1.06)	
	35–39	178 (2.04)	712 (2.04)	
	40–44	354 (4.05)	1416 (4.05)	
	45–49	570 (6.53)	2280 (6.53)	
	50–54	940 (10.76)	3760 (10.76)	
	55–59	1182 (13.53)	4728 (13.53)	
	60–64	1280 (14.66)	5120 (14.66)	
	65–69	1310 (15.00)	5240 (15.00)	
	70–74	1228 (14.06)	4912 (14.06)	
	75–79	801 (9.17)	3204 (9.17)	
	80–84	491 (5.62)	1964 (5.62)	
	85+	267 (3.06)	1068 (3.06)	
Sex, *n* (%)				0.00
	Male	5782 (66.21)	23,128 (66.21)	
	Female	2951 (33.79)	11,804 (33.79)	
Income, *n* (%)				0.00
	1 (lowest)	1702 (19.49)	6808 (19.49)	
	2	1141 (13.07)	4564 (13.07)	
	3	1416 (16.21)	5664 (16.21)	
	4	1849 (21.17)	7396 (21.17)	
	5 (highest)	2625 (30.06)	10,500 (30.06)	
Region of residence, *n* (%)				0.00
	Urban	3964 (45.39)	15,856 (45.39)	
	Rural	4769 (54.61)	19,076 (54.61)	
CCI score, mean (Sd)		0.77 (1.17)	0.66 (1.16)	0.10
Osteoporosis, *n* (%)		579 (6.63)	2909 (8.33)	0.07

Abbreviations: N/A, Not applicable; CCI, Charlson Comorbidity Index; Sd, standard deviation.

**Table 2:** Crude and adjusted hazard ratios (95% confidence interval) of colorectal cancer (CRC) for osteoporosis with subgroup analyses according to age, sex, income, region, and CCI scores.

		N of Event/ N of Total (%)	Follow-Up Duration (PY)	IR per 1000 (PY)	IRD (95% CI)	HR for Osteoporosis (95% CI)			
						Crude †	*p*	Adjusted ‡	*p*
Total participants (*n* = 43,665)									
	CRC	579/8733 (6.63)	41,967	13.80	−0.50 (−1.77–0.74)	0.96 (0.87–1.05)	0.33	0.95 (0.87–1.04)	0.302
	Control	2909/34,932 (8.33)	203,280	14.30		1		1	
Age < 65 years old (*n* = 23,180)									
	CRC	254/4636 (5.48)	25,573	9.93	1.17 (−0.10–2.45)	1.15 (1.00–1.32)	0.043 *	1.14 (0.99–1.31)	0.064
	Control	1065/18,544 (5.74)	121,590	8.76		1		1	
Age ≥ 65 years old (*n* = 20,485)									
	CRC	325/4097 (7.93)	16,394	19.80	−2.80 (−5.24–−0.25)	0.84 (0.75–0.95)	0.005 *	0.84 (0.75–0.95)	0.005 *
	Control	1844/16,388 (11.25)	81,690	22.60		1		1	
Male (*n* = 28,910)									
	CRC	141/5782 (2.44)	28,553	4.94	−0.33 (−1.25–0.59)	0.94 (0.78–1.12)	0.493	0.92 (0.77–1.11)	0.397
	Control	715/23,128 (3.09)	135,772	5.27		1		1	
Female (*n* = 14,755)									
	CRC	438/2951 (14.84)	13,414	32.70	0.20 (−3.19–3.49)	0.96 (0.87–1.07)	0.472	0.96 (0.87–1.07)	0.468
	Control	2194/11,804 (18.59)	67,508	32.50		1		1	
Low-income group (*n* = 21,295)									
	CRC	281/4259 (6.60)	19,337	14.50	0.40 (−1.39–2.28)	1.01 (0.89–1.15)	0.844	1.00 (0.88–1.14)	0.949
	Control	1354/17,036 (7.95)	96,120	14.10		1		1	
High-income group (*n* = 22,370)									
	CRC	298/4474 (6.66)	22,630	13.20	−1.30 (−3.06–0.37)	0.91 (0.80–1.03)	0.131	0.91 (0.80–1.03)	0.130
	Control	1555/17,896 (8.69)	107,160	14.50		1		1	
Urban resident (*n* = 19,820)									
	CRC	247/3964 (6.23)	19,971	12.40	−0.40 (−2.13–1.31)	0.96 (0.83–1.10)	0.515	0.95 (0.83–1.09)	0.502
	Control	1233/15,856 (7.78)	96,492	12.80		1		1	
Rural resident (*n* = 23,845)									
	CRC	332/4769 (6.96)	21,996	15.10	−0.60 (−2.41–1.21)	0.96 (0.85–1.08)	0.469	0.95 (0.85–1.07)	0.432
	Control	1676/19,076 (8.79)	106,788	15.70		1		1	
CCI scores = 0 (*n* = 27,426)									
	CRC	284/4887 (5.81)	23,703	12.00	−0.40 (−1.92–1.15)	0.95 (0.83–1.07)	0.397	0.96 (0.84–1.08)	0.485
	Control	1638/22,539 (7.27)	132,416	12.40		1		1	
CCI scores = 1 (*n* = 9119)									
	CRC	164/2262 (7.25)	10,812	15.20	−2.30 (−5.11–0.43)	0.84 (0.71–1.00)	0.048 *	0.90 (0.76–1.06)	0.215
	Control	700/6857 (10.21)	39,976	17.50		1		1	
CCI scores ≥ 2 (*n* = 7120)									
	CRC	131/1584 (8.27)	7452	17.60	−0.90 (−4.33–2.52)	0.92 (0.76–1.12)	0.421	1.01 (0.84–1.23)	0.880
	Control	571/5536 (10.31)	30,888	18.50		1		1	

Abbreviation: CRC, colorectal cancer; IR, incidence rate; IRD, incidence rate difference; PY, person-years; HR, hazard ratio; CI, confidence interval.† Stratified and unstratified Cox proportional hazard regression models were stratified by age, sex, income, and region of residence. ‡ Adjusted for CCI scores. * Significance at *p* < 0.05.

## Data Availability

All data are available from the database of National Health Insurance Sharing Service (NHISS) https://nhiss.nhis.or.kr/ (accessed on 1 October 2024). NHISS allows access to all of this data for any researcher who promises to follow the research ethics at some processing charge. If you want to access the data of this article, you can download it from the website after promising to follow the research ethics.
